# IDH mutation in glioma: molecular mechanisms and potential therapeutic targets

**DOI:** 10.1038/s41416-020-0814-x

**Published:** 2020-04-15

**Authors:** Sue Han, Yang Liu, Sabrina J. Cai, Mingyu Qian, Jianyi Ding, Mioara Larion, Mark R. Gilbert, Chunzhang Yang

**Affiliations:** Neuro-Oncology Branch, Center for Cancer Research, National Cancer Institute, Bethesda, MD 20892 USA

**Keywords:** Mutation, Cell biology

## Abstract

Isocitrate dehydrogenase (IDH) enzymes catalyse the oxidative decarboxylation of isocitrate and therefore play key roles in the Krebs cycle and cellular homoeostasis. Major advances in cancer genetics over the past decade have revealed that the genes encoding IDHs are frequently mutated in a variety of human malignancies, including gliomas, acute myeloid leukaemia, cholangiocarcinoma, chondrosarcoma and thyroid carcinoma. A series of seminal studies further elucidated the biological impact of the IDH mutation and uncovered the potential role of IDH mutants in oncogenesis. Notably, the neomorphic activity of the IDH mutants establishes distinctive patterns in cancer metabolism, epigenetic shift and therapy resistance. Novel molecular targeting approaches have been developed to improve the efficacy of therapeutics against IDH-mutated cancers. Here we provide an overview of the latest findings in IDH-mutated human malignancies, with a focus on glioma, discussing unique biological signatures and proceedings in translational research.

## Background

Isocitrate dehydrogenase (IDH) enzymes, of which there are three isoforms, are essential enzymes that participate in several major metabolic processes, such as the Krebs cycle, glutamine metabolism, lipogenesis and redox regulation.^1–3^ IDH1 is located in the cytoplasm and peroxisomes, whereas IDH2 and IDH3 are located in the mitochondrial matrix.^4^ The catalytic sites of IDH1 and IDH2 exhibit affinity for the substrate, isocitrate, together with nicotinamide adenine dinucleotide phosphate (NADP^+^) and a divalent metal cation, usually magnesium or manganese,^5^ resulting in the formation of α-ketoglutarate (α-KG). IDH3, which also catalyses the transformation from isocitrate into α-KG, employs nicotinamide adenine dinucleotide (NAD^+^) as its cofactor. The catalytic activity of IDH requires homodimerisation along with an alteration in the enzyme conformation; isocitrate binding changes the structure of the enzyme from an open to a closed conformation.^6^ Substrate recognition depends on the amino acid residues in the active site, whereas the frequent mutated active site residue in cancer is arginine 132 (R132).^5^

Mutations in IDH are prevalent in human malignancies. In glioma, IDH mutations are recognised in >80% of World Health Organisation (WHO) grade II/III cases.^7^ In WHO grade IV glioblastoma (GBM), IDH mutations are also found frequent in secondary GBM, which account for 73% of clinical cases, whereas they are less seen in primary GBM (3.7%).^8^ A follow-up investigation showed that the presence of IDH mutations predict a favourable disease outcome with prolonged median survival in GBM (IDH wild type: 15 months; IDH mutant: 31 months) and anaplastic astrocytoma (IDH wild type: 20 months; IDH mutant: 65 months).^7^ Although IDH-mutated glioma generally exhibits a better disease outcome, the high incidence of IDH mutations in secondary GBM suggests that lower-grade glioma with IDH mutation often recur with having undergone malignant transformation to a higher grade. In addition, IDH-mutated glioma is more likely to develop a hypermutation phenotype, which is associated with worsened prognosis.^9^ In non-central nervous system (non-CNS) malignancies, IDH mutations are identified in acute myeloid leukaemia (AML; 16% among all clinical cases),^10^ intrahepatic cholangiocarcinoma (23% among all clinical cases)^11^ and central/periosteal chondrosarcoma (56% among all clinical cases).^12^ The investigation of these non-CNS tumours with similar IDH mutation provides valuable information for glioma research, whereas in the present review we tend to be focussed on IDH-mutated glioma.

**Fig. 1:**
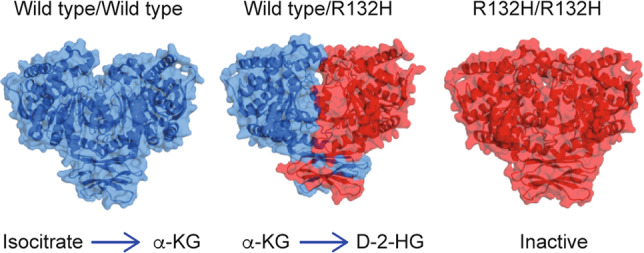
Dimerisation of IDH1. Two wild-type IDH1 monomers form a catalytic homodimer, which transforms isocitrate into α-KG. In IDH1-mutated cells, a catalytic heterodimer is formed with one wild-type monomer and one monomer carrying the R132H mutant. The heterodimer exhibits neomorphic activity, which consumes α-KG for D-2-HG synthesis. Biochemical studies indicate that a homodimer formed by two IDH1 mutant monomer is not catalytically active. Molecular modelling is based on the published crystal structures 1T09,^6^ 3MAS and 3MAP.^111^

IDH mutations that are associated with cancer tend to localise to the arginine residue that is crucial for the recognition of isocitrate (R132 for IDH1, R140 or R172 for IDH2).^7^ Missense mutations in the IDH1 gene result in the replacement of a strong, positively charged arginine residue at position 132 with lower-polarity amino acids such as histidine (H), lysine (K) or cysteine (C), which impedes the formation of hydrogen bonds with the α-carboxyl and β-carboxyl sites of isocitrate.^13,14^ The mutant IDH enzyme therefore exhibits decreased affinity for isocitrate, along with an elevated preference for NADPH. However, only one copy of the IDH gene is mutated in tumours and, in tumour cells harbouring heterozygous IDH mutations, the main forms of IDH dimers are presumed to be heterodimers that contain a version of wild-type IDH1 and a version with the R132H mutation. As a result, in IDH-mutant cells, the IDH1 wild-type component of the dimer converts isocitrate into α-KG to produce NADPH, whereas the mutant part of the dimer exhibits neomorphic activity, converting α-KG into D-2-hydroxyglutarate (D-2-HG) in an NADPH-dependent manner (Fig. 1).^15^

The establishment of neomorphic activity associated with the IDH mutation has long been shown to have an effect on cellular metabolism, cancer biology and oncogenesis.^16,17^ In this review, we will outline the biological impact of IDH-mutant neomorphic activity on cellular metabolism, epigenetic regulation and redox homoeostasis and address key advances in the cancer biology of IDH-mutated glioma and the implication of these findings in the development of future therapeutics, with a particular focus on glioma. Key findings from other IDH-mutated tumour models, such as AML and cholangiocarcinoma, will also be discussed, as they provide additional insights into the molecular mechanisms in IDH-mutated glioma.

## The biological impact of IDH-mutant neomorphic activity

### Metabolic reprogramming

IDH-mutant enzymes cause the accumulation of D-2-HG at concentrations as high as 5–30 mM^15^ in the cytoplasm, thereby draining carbohydrates from the Krebs cycle.^18^ The Krebs cycle is adjusted to compensate for fluctuations in the metabolic pathways.^19^ A ^13^C metabolic flux analysis suggested that IDH1-mutated cells exhibit increased oxidative metabolism in the Krebs cycle, whereas reductive glutamine metabolism is suppressed.^20^ With the depletion of cellular metabolism, several non-Krebs-cycle sources of carbohydrates are recruited to compensate for the loss of α-KG.^21,22^ Waitkus et al.^23^ demonstrated that glutamate dehydrogenase 2, an enzyme that catalyses the conversion of glutamate into α-KG and that is expressed at high levels in the brain, is important for relieving the metabolic liabilities in the context of IDH mutants. This finding is confirmed by the observation that IDH-mutated glioma cells are more sensitive to the inhibition of glutaminase,^24^ suggesting that glutaminolysis serves as a key compensatory pathway to maintain metabolic homoeostasis. McBrayer et al.^25^ further highlighted the dependency of IDH1-mutated cells on glutaminolysis, as D-2-HG functions as an inhibitor of the branched-chain amino acid transaminase (BCAT1/2), thereby decreasing the levels of glutamate. Furthermore, the consumption of NADPH by IDH mutants compromises de novo lipogenesis, resulting in an increased dependence on exogenous lipid sources for cellular growth.^2^ This is accompanied by the stimulation, by D-2-HG, of glutamine-derived lipogenesis under hypoxic condition to meet the needs for lipid productivity.^26^

Lactate dehydrogenase A (LDHA) catalyses the transformation of pyruvate formed by glycolysis into L-lactate,^27^ and the expression of LDHA is thus considered to be a hallmark of Warburg phenotype, allowing rapid glycolytic flux to meet the demands for cellular proliferation.^28^ Although LDHA is highly expressed in a variety of cancer cells, it is silenced in glioma tissue specimens and patient-derived glioma cells with IDH mutants.^29,30^ Silencing of LDHA (and of several other glycolysis genes including *CA9* and *VEGFA*) has been found to be associated with hypermethylation in the promoter region of these genes in response to D-2-HG. The overall epigenetic silencing of the glycolytic pathway might explain the slow-growing nature of IDH-mutated glioma as compared with their IDH wild-type counterparts.^30,31^ In support of this hypothesis, in a recent study, the acquisition of the Warburg phenotype was associated with more aggressive gliomas and was found to occur at the CpG island methylator phenotype (G-CIMP) in gliomas described below, which is specific for astrocytoma.^32^

In addition, IDH mutations lead to the neomorphic enzyme activity, which redirects the Krebs cycle for D-2-HG production. The resultant decrease in α-KG levels might affect the level of hypoxia-inducible factor subunit HIF-1α,^33^ as α-KG is normally needed for prolyl hydroxylases (PHD) to hydroxylate and promote the degradation of HIF. However, the detailed molecular mechanism on how HIF is regulated in the context of IDH mutation is currently unclear. Other lines of evidence showed that D-2-HG, but not L-2-HG, stimulates the activity of the prolyl hydroxylase PHD2, which results in the reduced expression of HIF-1/2α.^34^ More effort is encouraged to elucidate the relationship between D-2-HG and the hypoxia-sensing pathway in glioma and other IDH1-mutated malignancies.

**Fig. 2:**
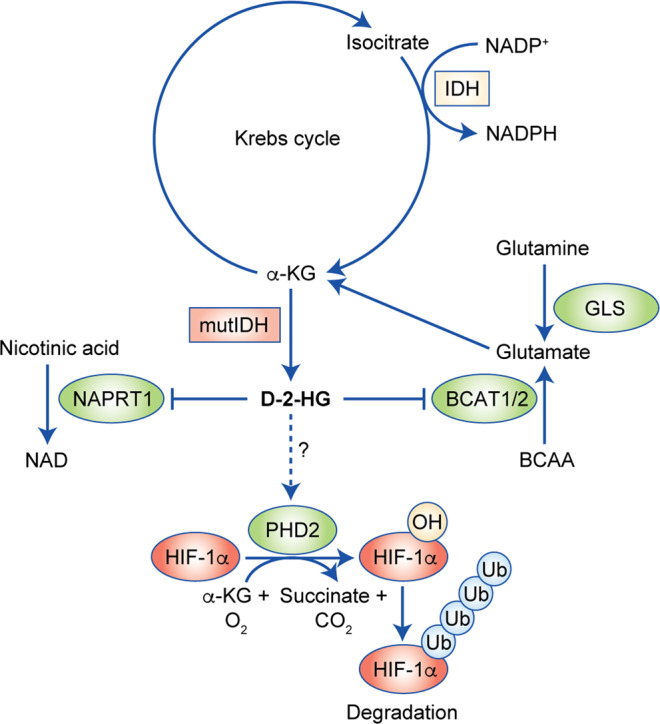
Metabolic reprogramming in IDH1-mutated glioma. Acquisition of IDH mutant results in substantial metabolic reprogramming. Neomorphic activity depletes the Krebs cycle by exhausting α-KG for D-2-HG production. Metabolites such as glutamine, glutamate and branched-chain amino acids (BCAA) serve as compensatory sources to fuel cellular metabolism. D-2-HG further impacts cellular metabolism such as the biosynthesis of glutamate and NAD. D-2-HG affects the biological function of PHD2, whereas the alterations in hypoxia-sensing pathway remain unclear.

Overall, the acquisition of mutant IDH results in substantial reprogramming of cellular metabolism (Fig. 2). Glutamine and/or glutamate serve as key substrates to compensate for the metabolic impact by strengthening synthetic pathways for lipids and glutathione. Interestingly, IDH-mutated glioma shows a distinctive metabolic pattern compared with other solid tumours—most notably, the remarkably reduced glycolysis, the metabolic hallmark of fast proliferating malignancies. The unique metabolic pathways in IDH-mutated glioma not only explain the slow-growing nature of this disease but also suggest that developing targeted strategies for IDH-mutant-specific metabolic patterns could be a valuable approach for future glioma therapeutics.

### Epigenetic reprogramming

**Fig. 3:**
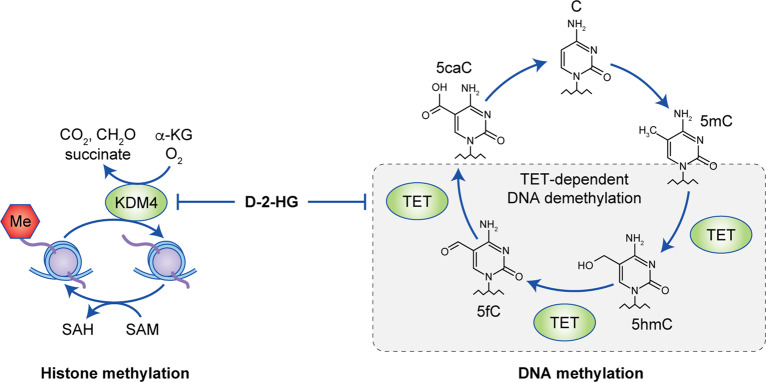
IDH1 mutants result in alterations throughout the epigenome. Owing to structural similarity, IDH1-mutant-derived D-2-HG serves as a competitive inhibitor for KDM4 or TET and therefore blocks the demethylation process in histone and nucleotide, respectively.

In addition to inducing metabolic alterations, the results of several clinical studies revealed that the IDH mutation is closely associated with CpG island hypermethylation. Indeed, the glioma-G-CIMP has been defined as a signature for IDH-mutated solid tumours.^35,36^ Mechanistic studies revealed that neomorphic IDH1 mutant activity results in both global DNA hypermethylation and histone methylation. Interestingly, the extent and targets of hypermethylation vary among tumour types.^37^ Investigation on the unique pattern of glioma-specific methylation pattern may assist the understanding of pathogenesis of IDH-mutated glioma (Fig. 3).

DNA methylation is controlled by methyltransferases and demethylases. Within the demethylation process, the enzyme Ten-eleven translocation methyl cytosine dioxygenase (TET) catalyses the conversion of 5-methylcytosine into 5-hydroxymethylcytosine (5-hmC) in an iron- and α-KG-dependent manner, as well as catalysing additional cytosine demethylation steps by transforming 5-hmC into 5-formylcytosine and 5-carboxylcytosine (5-caC). 5-caC will eventually be converted into cytosine by thymine DNA glycosylase and base excision DNA repair.^38^ The activity of TET is blocked by the presence of D-2-HG owing to its structural similarity with α-KG, which is indispensable for TET demethylase activity.^39,40^ In 2012, two research groups showed that acquisition of the cancer-associated IDH mutation is sufficient to induce a hypermethylation phenotype.^41,42^ Notably, although DNA methylation is generally believed to be a reversible process, a follow-up study suggested that some of the DNA methylation sites in IDH-mutated cells might persist even when the mutant enzyme is turned off, suggesting that the IDH mutation plays a key role in malignant transformation, which is irreversible once the cells have committed to oncogenesis.^43^

Besides DNA methylation, D-2-HG also increases methylation of histone by inhibiting histone demethylases such as lysine-specific demethylase (KDM).^39,44^ The methylation status of histone is regulated by histone methyltransferases, such as G9a, GLP, SET and EZH2, as well as by demethylases, such as KDM, LSD and JARID; similar to TET, histone demethylases such as KDM4 and KDM5 have been shown to be inhibited by high levels of D-2-HG.^44^ As a result, in IDH-mutated cancers, the accumulation of histone methylation markers such as H3K4me3, H3K9me3 and H3K27me3 is frequently reported. Inhibition of histone demethylation by D-2-HG results in impaired cellular differentiation, which might be relevant to the oncogenesis in IDH-mutated cancers.^45,46^

### Redox imbalance

**Fig. 4:**
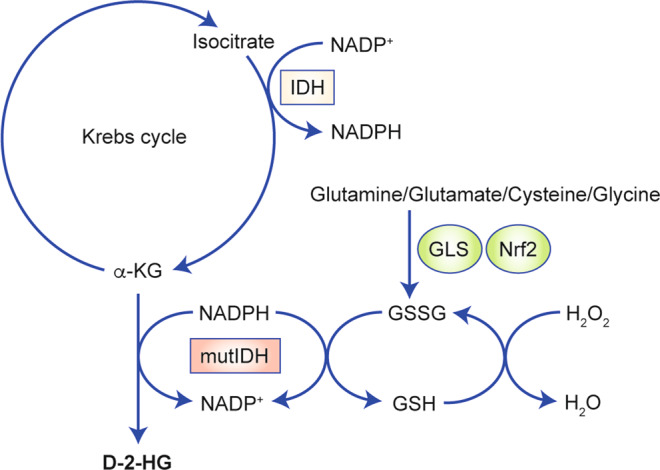
IDH mutants disrupt redox homoeostasis. The neomorphic activity of IDH1 mutant consumes NADPH for NADP^+^ production, which suppresses the detoxification of H_2_O_2_. Nrf2-associated gene transcription, such as glutamate-cysteine ligase (GLS), supports glutathione de novo synthesis. Amino acids such as glutamine, glutamate, cysteine and glycine are utilised for glutathione synthesis, which serves as an alternative metabolic support for ROS detoxification.

The production of reactive oxygen species (ROS) is involved in major aspects of cancer biology, such as genomic instability, loss of growth control, cellular motility and invasiveness.^47,48^ Excessive ROS is harmful to biological molecules, resulting in oxidative damage to DNA, lipids and proteins. Thus maintaining appropriate ROS levels is key to cancer cells during oncogenesis and therapeutic resistance.^49^ Cancer-associated IDH mutations elevate the affinity for both NADPH (*K*_m_ = 0.44 µM) and α-KG (*K*_m_ = 965 µM), indicating that the mutant IDH prefers NADPH and α-KG, instead of NADP^+^ and isocitrate, as its substrates.^15,16^ The consumption of cellular NADPH disrupts the reducing equivalents for biosynthetic reactions, which compromises key ROS-scavenging processes, such as glutathione disulfide reduction, leading to the accumulation of ROS.^50,51^ Consequently, accumulating oxidative damage is a hallmark of cancer biology for IDH-mutated malignancies (Fig. 4).^51,52^

Gilbert et al.^53^ showed that IDH1-mutated glioma cells exhibit strong oxidative stress, as evidenced by an enhanced expression of manganese superoxide dismutase and protein carbonylation. This increased stress was confirmed by subsequent investigations showing that IDH-mutated cancers are more prone to oxidative damage.^52,54^ We also confirmed elevated oxidative stress that is closely related to the acquisition of IDH mutants, leading to oxidative damage in biomolecules such as DNA and lipids.^51^ Owing to the substantially increased oxidative burden, inhibiting antioxidant pathways, such as the synthesis of glutathione, which is mediated by the transcription factor nuclear factor erythroid 2-related factor 2 (Nrf2), could be a valuable strategy for targeting IDH1-mutated solid tumours.^55^ In addition, proline synthesis has been reported to maintain redox homoeostasis in mitochondria in IDH1-mutated cells. Enhanced activity of pyrroline 5-carboxylate reductase 1-mediated glutamate-to-proline transformation in IDH-mutated cells alongside the oxidation of NADH partially uncouples the electron transport chain from Krebs cycle activity, thus maintaining anabolism in cancer cells.^56^

## Novel molecular targeting strategies for IDH-mutant glioma

### Direct targeting of mutant IDH

Given that the neomorphic activity of IDH mutants correlates with malignant transformation, direct targeting of the mutant enzyme has been a heavily pursued strategy. Rohle et al.^57^ reported the first synthetic inhibitor of the IDH mutant, AGI-5198, which blocks the production of D-2-HG and impairs IDH1-mutated xenograft growth in vivo. Second generation of IDH-mutant inhibitors, ivosidenib (AG-120) and vorasidenib (AG-881), are currently approved by the Food and Drug Administration as a therapeutic option for IDH-mutated AML.^58^ A good amount of clinical studies are currently completed/underway to evaluate the safety and efficacy of ivosidenib and vorasidenib for the treatment of IDH-mutated myeloid malignancies and solid tumours, including glioma.^59^ These IDH-mutant inhibitors exhibit an improved brain-to-plasma ratio, suggesting that they might be effective for IDH1-mutated glioma.^60^ Several other IDH-mutant inhibitors, such as BAY1436032, have shown tumour-suppressing effects as experimental therapeutics for the treatment of AML and astrocytoma in animal models.^61,62^ Two clinical studies (NCT03127735 and NCT02746081) are currently ongoing to confirm these findings in patients with IDH1-mutated AML or advanced solid tumours, respectively.

Despite the promising success of the IDH-mutant inhibitors, several studies have indicated the potential limitations of their application. For example, Johannessen et al.^63^ discovered that, although the IDH-mutant inhibitor AGI-5198 successfully reduces neomorphic activity, it relieves hypermethylation phenotype but to a much less extent, as evidenced by elevated histone-3 methylation. In addition, Sulkowski et al.^64^ reported that AGI-5198 relieves the burden of DNA damage in cancer cells, which might increase their resistance to genotoxic therapies, such as radiation and chemo agents. This phenomenon has been confirmed by another study showing that AGI-5198 confers radioprotective effects on IDH1-mutated cancer cells.^65^ Overall, targeting IDH-mutant neomorphic activity is a straightforward strategy and has shown efficacy against haematopoietic malignancies in humans and several experimental models for solid cancers. In addition to suppressing D-2-HG production, a combined approach with other agents, such as inhibitors of critical enzymes in metabolic or DNA repair pathways, might be helpful to improve the disease outcome (see the discussion below on synthetic lethality).

### Epigenetic modulators

As outlined above, the IDH mutation induces a hypermethylation phenotype that results in broad alterations to the epigenetic landscape in tumour cells.^41,43,66^ Importantly, the hypermethylation phenotype might be linked with oncogene activation. Correcting the epigenetic dysregulation has thus been advocated as a potential therapeutic strategy for cancers that express the IDH mutant. Flavahan et al.^67^ discovered that the G-CIMP profile correlates hypermethylation at binding sites for cohesion and CCCTC-binding factor (CTCF) and therefore compromises the affinity of this protein. The loss of CTCF binding allows an enhancer to mediate the constitutive expression of platelet-derived growth factor receptor A (PDGFRA), a known mitogen that is linked with glioma oncogenesis. Administration of a demethylating agent partially restores CTCF binding, leading to reduced PDGFRA expression. The concept of inhibiting methylation was confirmed through another study, which demonstrated that decitabine, a DNA methyltransferase (DNMT1) inhibitor, suppresses the proliferation of IDH-mutated glioma cells in vitro and in vivo.^68^ Similarly, 5-azacytidine, a cytidine analogue that compromises the activity of DNA methyltransferase, resulted in the regression of a patient-derived IDH1-mutated glioma xenograft.^69^ However, although epigenetic modulators might sufficiently reverse the IDH-associated hypermethylation phenotype, their effects on other cancer signatures in IDH-mutated tumours, such as metabolic reprogramming and DNA repair pathways, remain unclear.

### Targeting DNA repair enzymes

IDH-mutated glioma exhibits a favourable disease outcome compared with its wild-type counterpart. Several lines of evidence suggested that glioma with mutant IDH appeared to be more sensitive to treatment standards of care, such as radiotherapy and chemotherapy, which may explain their preferred disease outcome.^70–72^ Mechanistically, D-2-HG compromises DNA repair pathways: it serves as an inhibitor to DNA repair enzymes such as AlkB homologue 2/3 (ALKBH2/3),^73,74^ as well as inhibiting the homologous recombination (HR) DNA repair process.^64^ With the general DNA repair pathways inhibited, targeting the remnant DNA repair enzymes could be detrimental for IDH-mutated cells and thus provide a potential therapeutic approach. We and several colleagues showed that a combination of small-molecule inhibitors targeting the poly-ADP ribose polymerase (PARP) could be a highly effective therapy for IDH-mutated malignancies.^64,75,76^ Owing to the suppression of HR pathways, IDH-mutated cells resemble serious DNA repair defects, which are commonly seen in cancers with *BRCA* mutations (“BRCAness”). Cancer cells develop a dependency on PARP-guided base excision DNA repair (BER), which helps to maintain genomic integrity under genotoxic therapy.^64,77,78^ PARP inhibitors can establish synthetic lethality with IDH-mutant-derived HR deficiency, resulting in enhanced apoptotic changes.^79^ Similarly, Tateishi et al.^80,81^ showed that the inhibitors of nicotinamide phosphoribosyltransferase (NAMPT) FK866 and GMX1778 deplete NAD^+^, thereby eliminating remaining PARP DNA repair activity (as PARP requires NAD^+^ during BER of chemotherapy-induced DNA damage), which introduces distinct metabolic stress responses to chemotherapy-induced DNA damage and improves the durability of therapy response.

Although the acquisition of the IDH mutation correlates with therapeutic sensitivity in patients and targeting DNA repair enzymes seems to be more effective for IDH-mutated cells, several investigations have highlighted that IDH-mutated glioma might develop distinctive DNA repair pathways from those of IDH wild-type glioma. For example, RAD51 recombinase, an enzyme that plays major roles in the HR process, protects IDH-mutated cells from temozolomide-induced DNA damage.^82^ Nunez et al.^83^ showed that the depletion of TP53 or ATRX on an IDH-mutated background resulted in glioma cells tending to undergo a DNA damage response, highlighted by upregulated ATM signalling and resistance to radiotherapy. Taken together, the discovery of altered DNA repair pathways in IDH-mutated glioma not only highlights the correlation between cancer metabolism and genomic instability but also implies important therapeutic vulnerabilities in cancers with IDH mutation.

### Targeting essential metabolic enzymes

The substantial reprogrammed cellular metabolism in IDH-mutated glioma suggests that it might be possible to establish specific druggable targets for this type of malignancy. NAD, a cofactor for critical biological processes such as electron transport and redox metabolism, is derived from biosynthesis (de novo pathway) and salvage pathways (using compounds containing a pyridine base).^84^ In IDH-mutated glioma, the de novo synthesis of NAD is largely compromised owing to epigenetic silencing of nicotinate phosphoribosyltransferase (NAPRT1). As a result, cancer cells rely on the salvage pathway to generate NAD.^81,85^ The dysfunction in NAD metabolism suggests that IDH-mutated cells could be extremely sensitive to the blockade of the salvage pathway through small-molecule inhibitors targeting NAMPT.^81^

In addition, owing to the essential role of glutaminolysis in metabolic compensation, targeting glutamine/glutamate-related metabolism has been proposed for IDH-mutated malignancies. For example, glutaminase blockade through bis-2-[5-(phenylacetamide)-1,3,4-thiadiazol-2-yl]ethyl sulfide inhibits glutamine metabolism and suppresses IDH1-mutated glioma and AML.^24,86^ In light of high-throughput screening assays, Zaprinast was found to inhibit glutaminase and limited the proliferation of IDH-mutated tumour cells in vitro.^87^ CB-839 is an oral glutaminase inhibitor that has shown therapeutic efficacy in IDH-mutated AML, causing a reduction in D-2-HG production and inducing terminal differentiation.^88^ A phase 1 clinical study is currently underway to evaluate the combination of CB-839, radiation and the alkylating chemotherapy agent temozolomide in IDH-mutated diffuse or anaplastic astrocytoma (NCT03528642). Overall, suppressing glutamine/glutamate metabolism correlates with slowed tumour manifestation in both glioma and AML models. However, the strategy commonly results in prolonged latency rather than strong tumour suppression in vivo. Combining the suppression of glutamine/glutamate metabolism with other cytotoxic therapies, such as radiation and temozolomide, might improve the therapeutic efficacy.

### Targeting redox homoeostasis

As mentioned previously, elevated ROS is a hallmark of IDH-mutated malignancies.^53,89,90^ An imaging study demonstrated that levels of glutamate, glutamine and glutathione are decreased in tumour regions in patients with IDH-mutated glioma, as compared with levels in contralateral regions. In addition, the tumoural glutathione level negatively correlates with the level of D-2-HG, suggesting that glutathione is essential for IDH-mutated cells to maintain redox homoeostasis.^91^ The increased consumption of glutathione suggests a high burden of ROS scavenging in IDH-mutated cells. As such, developing a therapeutic strategy to disrupt the fragile redox homoeostasis, such as limiting glutathione-derived ROS scavenging, could be a valuable therapeutic approach. A preclinical animal study has shown that inhibiting glutamine metabolism using the glutaminase inhibitor CB-839 leads to impaired redox homoeostasis and sensitises IDH-mutated glioma to radiotherapy.^25^ In our studies,^51,55,92^ we have shown that suppression of Nrf2 by the natural compound brusatol resulted in profound tumour suppression in IDH1-mutated xenografts, along with overwhelming oxidative damage. Although disruption of redox homoeostasis in these cases results in potent cytotoxicity accompanied by tumour suppression, current therapeutic compounds are mostly at the preclinical stage and demonstrate considerable systemic toxicity. Nevertheless, developing the next generation of therapeutic compounds with both potency and selectivity will be of great help for targeting redox imbalance in IDH-mutated malignancies.

### Immunotherapies

There is growing evidence that the IDH mutation might play important roles in altering the tumour immunological microenvironment, as indicated by a suppression of tumour-infiltrating lymphocytes, natural killer cells and cytotoxic T cells.^93,94^ An initial investigation on glioma data sets showed that the presence of IDH mutation correlates with a decrease in the expression of programmed death-ligand 1 (PD-L1)^95^ when compared with IDH wild-type gliomas. Reduced PD-L1 expression in IDH-mutated glioma implies a stronger T cell activation, as PD-L1 is a cellular surface protein that downregulates the immune system and promotes self-tolerance through suppressing T cell activity.^96^ In addition, D-2-HG-derived DNA methylation results in epigenetic silencing of both PD-1 and PD-L1 in glioma.^97,98^ However, the reduced expression of PD-1/PD-L1 in IDH-mutated glioma may not result in stronger antitumour T cell immunity. An in-depth investigation showed that D-2-HG serves as a potent inhibitor for antitumour T cell immunity in the tumour microenvironment. D-2-HG suppresses ATP-dependent T cell receptor signalling, which further impairs the activation of T cells in glioma. D-2-HG also suppresses signal transducer and activator of transcription 1, leading to decreased CD8^+^ T cell recruitment in the tumour region.^99^ Combining a pan-IDH1-mutant inhibitor BAY1436032 improves the efficacy of anti-PD-1-derived immunotherapy, causing enhanced intratumoural CD4^+^ T-cell proliferation, a reduction in tumour volume and prolonged overall survival.^94^ Additional immune checkpoint inhibitors are currently under investigation for IDH1-mutated glioma. The PD-L1 inhibitor pembrolizumab (MK-3475) is being evaluated in a clinical trial of patients with recurrent IDH1-mutant grade II–IV gliomas whose tumours have a hypermutator phenotype (NCT02658279). Another ongoing phase 1 clinical trial is currently enrolling patients with IDH1-mutant gliomas that have transformed into GBM to investigate the concurrent administration of the PD-L1 inhibitor avelumab and hypofractionated radiation therapy (NCT02968940).

Despite PD-1-derived immunotherapy, several attempts have been made to generate a peptide vaccine that targets the IDH1 R132H neoantigen. An IDH1 R132H-specific immunogenic epitope was presented by major histocompatibility complex (MHC) class II molecules and induced a CD4^+^ T_H_1 response, resulting in an effective MHC class II-restricted antitumour immune response in tumours with the IDH1 R132H mutation.^100^ Consistent with this result, another study found that mice immunised with peptides encompassing the IDH1 mutation site showed increased survival benefit when bearing GL261 gliomas with IDH1 R132H expression but not parental GL261 gliomas. The immunised mice showed higher amounts of peripheral CD8^+^ T cells and produced higher levels of interferon-γ, indicating the generation of anti-IDH1-mutant antibodies.^101^ The IDH-mutant vaccine is currently being investigated in clinical studies. For example, a phase 1 clinical trial is currently ongoing to validate the safety and therapeutic efficacy of an IDH1 R132H mutant peptide vaccine (NOA-16) in newly diagnosed grade III and IV gliomas with an IDH1 mutation (NCT02454634). The first reported results demonstrated the safety and immunogenicity of NOA-16, with 80% of patients displaying mutation-specific T cell immune responses, and 87% of the patients displaying humoural immune responses; no deaths were reported.^102^

## Conclusions and future perspectives

The discovery of the IDH mutation not only adds to the landscape of glioma genetics but also indicates that glioma is a highly heterogeneous disease. Many pioneering studies have shown that different glioma molecular subtypes exhibit different signatures according to their oncogenic drivers and distinctive patterns of therapy resistance. For example, amplifications or gain-of-function mutations in the genes encoding epidermal growth factor receptor and PDGFRA are highly common in GBM.^103^ The loss of phosphatase and tensin homologue compromises the HR DNA repair pathway, which predisposes to sensitivity to temozolomide and PARP inhibitors.^104^ For IDH-mutated gliomas, numerous attempts have been made to define selective and effective therapeutics that target the biological signatures of IDH-mutated cancers, with the aim of improving standard treatments. For instance, the addition of PARP inhibitors or blockade of DNA repair enzymes improves the cytotoxicity of genotoxic therapies. Targeting distinctive metabolic patterns such as glutaminase and glutathione de novo synthesis has also shown potent efficacy in IDH1-mutated cells.^105^ However, a major hurdle in IDH1-mutated glioma is that the critical oncogenic drivers of this disease remain controversial. Increasing evidence has uncovered the biological impact of D-2-HG on cancer biology, but the molecular targets of D-2-HG includes >60 members,^38,106^ which are involved in profoundly diverse molecular pathways, so the identification of clear oncogenic mechanisms in IDH-mutated glioma remains challenging. In-depth investigation into the critical molecular pathways will be of great importance to develop therapeutic approaches with high potency and selectivity.

Cancer therapeutics that exploit the concept of synthetic lethality by targeting multiple biologically relevant molecular pathways are expected to have reduced toxicity, as they tend to be more specific to cancer cells due to their unique mutation pattern. IDH-mutated glioma exhibits clear dysfunctions in several biological pathways. For example, metabolic depletion through IDH-mutant neomorphic activity prompts dependency on alternative metabolic pathways, such as glutaminolysis and NAD salvage pathways. Targeting critical enzymes, such as glutaminase or NAMPT, establishes synthetic lethality with the intrinsic metabolic dysfunction and translates into reduced tumour expansion. Moreover, D-2-HG inhibits DNA repair pathways such as HR and PARP/BER, which confers sensitivity to inhibition of the remaining DNA repair enzymes. Targeting mechanisms of ROS scavenging synergises with IDH-mutant-derived oxidative stress, which improves disease outcome with reduced IDH1-mutated xenograft growth. Taken together, targeting the distinctive vulnerabilities of IDH-mutated glioma has been shown to be successful, as cancer cells are less likely to compensate for the loss of essential biological pathways. Moreover, the vulnerabilities in IDH-mutated cells such as glutaminolysis and ROS scavenging are generally absent in other somatic cells, suggesting that the approach of synthetic lethality might be better tolerated in the context of combination therapy.

The development of disease models with biological and clinical relevance has become one of the major challenges for basic and translational research for IDH1-mutated glioma. Cell lines stably expressing mutant IDH1, which have long been used in many studies, are powerful tools for investigating the biological consequences of IDH mutants in isogenic backgrounds. However, the parental cells are commonly derived from GBM or other malignant tumours, which harbour genetic abnormalities that are rarely seen in lower-grade glioma, so it is very hard to justify the application of using these cell lines to investigate IDH1-mediated de novo oncogenesis and malignant transformation. Furthermore, establishing sustainable cell strains with intrinsic IDH mutation has been challenging, especially in cases of low pathological grades. Several studies have reported that IDH-mutated glioma cell lines are useful for in vitro investigations, but they have limited application in in vivo experiments due to the extremely low efficiency of xenograft formation (Table 1). Moreover, several investigations have suggested that IDH-mutated cells develop additional genetic alterations after subculture. For example, the BT142 cell line, an anaplastic oligoastrocytoma derived from brain tumour stem cells, carries both the wild-type and mutant IDH1 allele;^107^ however, the wild-type allele is gradually lost after passaging the cells in vitro.^108^ As another example, Mazor et al.^109,110^ discovered that patient-derived glioma cells with IDH mutations could undergo hemizygous deletion of the IDH1 locus, suggesting that tertiary mutations might have been established to enable cells to be sustained in vitro.^109,110^ IDH-mutated patient-derived glioma cells might therefore provide useful information for mechanistic studies, but any conclusions should be carefully verified in further preclinical models. Genetically engineered mouse models have the potential to provide valuable tools for investigating IDH1-mutated glioma. Several emerging mouse models provide pivotal tool to understand the cancer biology in IDH-mutated glioma (Table 2). These genetically engineered mice provide highly biologically relevant, powerful preclinical models; the resulting tumours were mostly of high pathological grades. Modelling low-grade tumours will be invaluable in order to gain additional insights into IDH-mutated glioma.

**Table 1:** Xenograft models for IDH-mutated glioma.

Mouse strain	Cell line	IDH mutation status	Applications	References
NOD SCID mice, nude mice	TS603	Intrinsic IDH1^R132H^	Subcutaneous xenograft	^57,117^
NOD SCID mice, nude mice	BT-142	Intrinsic IDH1^R132H/−^	Intracranial xenograft: MS 93–112 days	^107,118,119^
NOD SCID mice	MGG-(60, 79, 88, 108, 117, 119, 132, 152)	Intrinsic IDH1^R132H^	Intracranial xenograft	^81,120^
NOD SCID mice	GB10	Intrinsic IDH1^R132H^	Intracranial xenograft	^119^
NOD SCID mice, nude mice	HT1080	Intrinsic IDH1^R132C^	Subcutaneous xenograft	^87^
NSG mice	Inducible IHAs, IDH1 R132H-expressing (Dox+) tumour	Transduced IDH1^R132H^	Intracranial xenograft	^43^
ICR SCID mice	HOG-R132H	Transduced IDH1^R132H^	Intracranial xenograft HOG-EV, MS ~15 days; HOG-R132H, MS ~13 days	^25,42^
SCID SHO mice, nude mice	U87MG-R132H	Transduced IDH1^R132H^	Intracranial xenograft, MS 40 days; Subcutaneous xenograft	^121^
SCID SHO mice	HCT116-R132H	Transduced IDH1^R132H^	Subcutaneous xenograft	^122–124^
SCID SHO mice	GBM164	Intrinsic IDH1^R132H^	Subcutaneous xenograft	^122,125^
Nude mice	HeLa cells (with and without IDH1 mutation)	Transduced IDH1^R132H^	Subcutaneous xenograft	^124^
Nude mice	JHH-273 tumour	Intrinsic IDH1^R132H^	Subcutaneous xenograft	^69^
Nude mice	NCH551b	Intrinsic IDH1^R132H^	Intracranial xenograft	^61^
C57BL/6 mice	GL-261-MUT	Transduced IDH1^R132H^	Intracranial xenograft, MS 21.5 days	^99^

*MS* median survival, *NOD* non-obese diabetic, *SCID* severe combined immunodeficiency.

**Table 2:** Transgenic mouse models for IDH-mutated glioma.

Mouse strain	DNA/virus construct	D-2-HG	Incidence	Median survival	References
Ntva, Ink4a-Arf^−/−^	RCAS-PDGF RCAS-IDH-H1-shp53	~100-fold	NA	WT: 36 days R132H: 35 days	^112^
Ntva, Cdkn2a^f/f^, Pten^f/f^, ATRX^f/f^	RCAS-Cre RCAS-PDGFA RCAS-IDH1-R132H	~100-fold	WT: 20% R132H: 88%	WT: 150 days R132H: 43.5 days	^113^
Ntva, Cdkn2a^f/f^	RCAS/luc–PDGFB RCAS/YFP–IDH1^R132H^; RCAS/IDH1^R132H^–PDGFB	3583 nmol/mg protein	WT: 0% R132H: 93%	WT: NA R132H: 43 days	^114,115^
C57BL/6	pT2C-LucPGK-SB100X, pT2-shp53-GFP4, pT2CAG-NRASV12, pT2-shATRX53-GFP4, pKT-IDH1(R132H)-IRES-Katushka	8.16 μg/mg of protein	NA	WT: 70 days R132H: 163 days	^83^
Idh1LoxP^R132H^/+	Retrovirus PDGFB-IRES-CRE	Elevated	>90%	WT: 29 days R132H: 34 days	^116^

*NA* not applicable, *WT* wild type.

In summary, mutations in IDH are clearly linked to the establishment of human malignancies. A series of seminal studies have revealed the impact of IDH mutant and D-2-HG in cellular physiology, such as reprogrammed metabolism, epigenome alterations and redox homoeostasis. With the increased availability of disease models both in vitro and in vivo, more breakthroughs are anticipated to elucidate the critical pathways that are involved in tumour formation, tumour metabolism and therapeutic vulnerability. A molecular targeting and synthetic lethality approach would become available to benefit patients with IDH-mutated glioma, with improved disease outcome and quality of life.

## Data Availability

Not applicable.
